# Physical fitness characteristics and performance in single-handed dinghy and 470 classes sailors

**DOI:** 10.1016/j.jesf.2021.11.001

**Published:** 2021-11-11

**Authors:** Dandan Pan, Bingshu Zhong, Wei Guo, Yixiao Xu

**Affiliations:** a Institute of Physical Education and Training, Capital University of Physical Education and Sports, 100191, China; b School of Physical Education and Sport Training, Shanghai University of Sport, 200438, China

**Keywords:** Sailing, Single-handed dinghy, 470 sailors, Physical fitness, Performance

## Abstract

The purpose of this study was to investigate the differences in the physical fitness characteristics of Chinese single-handed dinghy and 470 sailors, predict the single-handed dinghy sailors’ physical factors on the performance by equation to guide the training. The sample of this study consisted of one hundred and sixty-seven Chinese sailors who participated in the 2020 China National Sailing Championships, K Independent Samples Test was used to analyze the differences of different classes sailors, and the performance of Laser and Laser Radial sailors were analyzed by step multivariate linear regression. The results showed that the 470 helmsmen are shorter, lighter and have a lower BMI, 470 crew are similar in height to the single-handed dinghy sailors, but lower in weight and BMI. Laser sailors have better strength and flexibility than the ones of male 470. There is no significant difference in the physical fitness characteristics between the Laser Radial sailors and the female 470 crew, they both had better upper and lower body strength than female 470 helmsmen. The regression equation is possible to explain 65.5% of the performance of the sailors in Laser = 90.963-1.33 × sailing experience-0.461 × bench press-0.018 × cycling peak power out; The regression equation is possible to explain 76.7% of the performance of the sailors in Laser Radial = 27.433-0.391 × sailing experience+0.351 × vertical jump-0.329 × pull-up-0.027 × cycling peak power out. Performance in laser and Laser Radial sailors will be determined by the technique and tactics (sailing experience) as well as physical fitness.

## 1 Introduction

Sailing is a sport driven by wind, sailors constantly adjust their sails and hull posture to keep balance and promote the boat move forward at high speed. The performance of the race is affected by many factors, such as anthropometry characteristics, physical fitness, skills and tactics.^1^ Olympic sailing regattas consist of two to three races per day for 5–7 days, as the format of the race has changed, the duration of each match is relatively short, and high-level physical exertion is required.^2^ Laser and Laser Radial sailors are single-handed dinghy, 470 sailors are double-handed dinghy which include helmsmen and crew, the differences boat type and motions needed may reflect the sailors’ physical requirements. Bojsen-Møller classifies sailors according to their body position during the race, single-handed dinghy sailors and 470 helmsmen are “side-deck hikers” (sailors are sitting on the deck and leaning over the side with their feet fixed), 470 crew are trapeze sailors (sailors stand on the gunwale or side swings of the boat supported by a wire that is extended from the rigging).^3^^,^^4^

The characteristics of different movement patterns compared to a sailing class and position, therefore there is a large variability of anthropometry and physical demands in sailors. Anthropometry characteristics define the structure of sailors and have a direct connection with biomechanical outputs, and physical fitness elements are related to economy and efficiency of movements, which are important factors in optimizing sailors' performance.^5^, ^6^, ^7^ As we known, the single-handed dinghy sailors greatest exerted physical maneuver is hiking, which needs strong strength and endurance of quadriceps as well as abdominal muscles.^8^ The physical demands of 470 sailors are most reflected in pumping maneuver, which involves a rapid flexion-extension movement of the spine. Pumping the sail in an effort increases muscle activations and dynamics of sailing, causing higher oxygen consumption.^9^ In addition, sailing performance is also affected by flexibility, power, aerobic and anaerobic capacity, which are incorporated into the sailor's physical training program along with strength and endurance.^4^^,^^10^^,^^11^

At present, information is scarce regarding the physical fitness of different sailing classes, and we also don't know which factors have a greater impact on a sailors' performance, it is detrimental to the long-term development of sailing. In this study, one hundred and sixty-seven Chinese sailors participated in the 2020 National Sailing Championships, including single-handed dinghy and 470 classes (Laser, Laser Radial, 470 man and woman, 470 mixed). The purpose of this study was to: (1) understand the physical fitness characteristics and differences in single-handed dinghy as well as 470 sailors. (2) discuss the influence of physical factors on the performance of single-handed dinghy sailors, and predict the performance by equation to help us formulate physical training program.

## 2 Materials and methods

### 2.1 Participants

One hundred and sixty-seven sailors participated in the study. There are ninety-three male sailors (38 Laser sailors, 26 helmsmen, 29 crew) and seventy-four female sailors (27 Laser Radial sailors, 25 helmsmen, 22 crew). All sailors underwent a systematic medical examination to exclude patients with major diseases. The content, process and potential risks of the study were explained to all the participants, who are voluntarily joined this study with informed consents. The study protocol was approved by the Capital University of Physical Education and Sports Ethics Committee and according to the ethical principles of the World Medical Association Declaration of Helsinki.

### 2.2 Measurements and procedures

The physical tests were performed before 3 days regatta at the Qinhuangdao Sailing Training Base, the anthropometric measurements were recorded. In first day, anthropometric measurements, nonfatiguing tests (height, weight, sit-and-reach, vertical jump) and anaerobic capacity test (30-s sprint cycle test) were assessed. In second day, muscular strength (back squat, bench press) and strength endurance tests (pull-up) were performed. In last day, aerobic capacity was tested (3 km run). Wind conditions was tested in real time during the regatta.

#### 2.2.1 Anthropometry

The measurements were Height (cm), weight(kg), BMI (kg/m^2^), and the test tools were height meter and weight scale. The tests were completed by specialized personnel of Hebei Sports Bureau in strict accordance with the detailed rules of “Sports Measurement and Evaluation".

#### 2.2.2 Flexibility test (sit-and-reach)

The sailors sat shoeless on the Sit-and-reach tester (LK-T5016, LINGKANG, China) with the feet separated approximately hip-wide and the toes pointed upward, kept the hands adjacent to each other and not lead with one hand, slowly reached forward as far as they could by sliding their hands along the measuring board.^12^ The tester pressed the athlete's knees down and straighten. The best of three trials were recorded.

#### 2.2.3 Power test (vertical jump)

The sailors performed vertical jump using wireless jump test system with counter-movement vertical jump on a mat that was attached to a data collection computer (EZEJUMP, SWIFT, Australia). Jumps were performed with hands on hips, which hands-on-hips method was adopted in order to concentrate on leg and hip explosiveness and minimize jumping technique differences.

The best of three attempts was recorded and the vertical jump height was computed.^13^^,^^14^

#### 2.2.4 Anaerobic capacity (30-s sprint cycle test)

The sailors’ anaerobic capacity (peak and mean power output) was performed on an air-braked cycle ergometer (Wattbike Pro, Nottingham, UK), which calculates power output by measuring the chain tension over a load cell. Before the test, the athletes performed routine warm-up, and carried out 1 incremental load cycling and 2 short sprints (3-s maximal sprints with 20-s of easy pedaling between) on the bicycle. The test was conducted after a 60-s rest. All subjects were instructed to perform the sprint as a maximal all-out effort with a target power output relative to their individual body weight, the computer attached to the cycle ergometer was used to record 30-s mean and peak power output during the sprint test.^15^

#### 2.2.5 Strength tests (back squat and bench press)

The strength test process was carried out according to the method recommended by the National Strength & Conditioning Association.^16^ Familiarization was conducted through a self-determined, exercise-specific warm up typically consisting of 3–4 sets of the particular exercise with submaximal loads, beginning with a relatively light one. The first warmed up using 50% of the athlete's estimated 1RM (the 1-repetition maximum) weight. After rested 1–5 min, the strength and conditioning professional increases the weight by 5–10% in bench press and 10–20% in back squat. The 1RM was determined within six attempts.

#### 2.2.6 Strength endurance test (pull-up)

The sailors hold the bar with their forehand and arms outstretched as the starting position, then pulled their body up until lower jaw was above the horizontal bar. Returning to the fully extended suspension position on each repeat. The sailors can't make any unnecessary movements, including excessive body sway.^17^

#### 2.2.7 Aerobic capacity (3 km run)

In a 400 m track, all sailors should line up behind the starting line. On an auditory signal, the sailors started running and covered the course as quickly as possible. As the runners cross the finish line, each sailor's time was recorded.

#### 2.2.8 Regatta wind conditions

Wind tester (Windbot, YachtBot, New Zealand) was used for the wind intensity of the races and calculated true wind information. The wind speeds in the 2020 National Sailing speed Championship range from 6.66 to 15.57 knots in single-handed dinghy races and from 6.97 to 15.53 knots in 470 class races.

### 2.3 Statistical analyses

Statistical analysis was performed with the SPSS Statistics V26.0 software. The mean and standard deviation of the data are represented. The differences between single-handed dinghy and 470 sailors were analyzed with the Kruskal-Wallis H. We set the significance level at *P* < 0.05. Different genders were divided into three groups, male group included: Laser sailors, 470 Helmsmen, and 470 crew, female group included: Laser Radial sailors, 470 Helmsmen, and 470 crew. A multiple linear regression analysis was carried out with the aim of examining the association between testing parameters and single-handed dinghy sailors’ performance, considering the following as predictive variables: sailing experience, back squat, bench press, vertical jump, sit and reach, pull-up, cycling peak power out, cycling mean power output, 3 km run. The forward stepwise regression method was used to perform the multiple linear regression analysis.

## 3 Results

Table 1 is the demographic information of sailors. In both male and female groups, 470 helmsmen were shorter, lighter and had a lower BMI than other sailors. The height of male 470 crew was similar to Laser sailors, but the weight and BMI were significantly lower (*p* < 0.01). Laser Radial sailors were shorter than female 470 crew (*p* < 0.01).

**Table 1:** Demographic information of sailors.

	Male			Female		
	Laser (N = 38)	470 Helmsmen (N = 26)	470 Crew (N = 29)	Laser Radial (N = 27)	470 Helmsmen (N = 25)	470 Crew (N = 22)
Age (years)	20.55 ± 3.8	21.12 ± 5.96	21.31 ± 4.08	20.48 ± 4.37	20.72 ± 5.86	20 ± 4.39
Sailing experience (years)	6.79 ± 4.68	9.04 ± 5.4	6.28 ± 3.97	6.52 ± 5.49	8.28 ± 5.44	5.55 ± 4.39
Height (cm)	182.08 ± 5.04	172.32 ± 4.24∗	183.59 ± 4.71^#^	171.43 ± 3.62	163.24 ± 5.63^+^	177.42 ± 3.66^+※^
Weight (kg)	76.26 ± 4.69	60.18 ± 5.29∗	71.91 ± 5.92∗^#^	60.82 ± 4.29	50.59 ± 4.08^+^	63.95 ± 3.66^※^
BMI（kg/m^2^）	23.01 ± 1.34	20.26 ± 1.55∗	21.35 ± 1.82∗	20.7 ± 1.33	18.99 ± 1.29^+^	20.31 ± 1.26^※^

Note: In male group, ∗significant difference compared with the Laser sailors (*p* < 0.01), ^#^ significant difference compared with the male 470 helmsmen (*p* < 0.01).

In female group, ^+^significant difference compared with the Laser Radial sailors (*p* < 0.01), ^※^ significant difference compared with the female 470 helmsmen (*p* < 0.01).

Table 2 shows the results of the physical fitness tests in all sailors. The sit-and-reach, bench press and back squat strength of Laser sailors were better than the ones of the male 470 sailors (*p* < 0.01). The repetitions of pull-up of male 470 helmsmen were significantly more than the ones of other sailors (*p* < 0.05). Female 470 crew sailors’ bench press and back squat strength was significantly better than female 470 helmsmen (*p* < 0.05).

**Table 2:** The results of the physical fitness tests in all sailors.

	Male			Female		
	Laser (N = 38)	470 Helmsmen (N = 38)	470 Crew (N = 38)	Laser Radial (N = 27)	470 Helmsmen (N = 25)	470 Crew (N = 22)
Bench press (kg)	91.3 ± 8.53	73.99 ± 9.13∗∗	83.92 ± 9.89∗∗^##^	55.85 ± 8.9	49.44 ± 7.9^+^	61.21 ± 11.21^※※^
Back squat (kg)	125.39 ± 15.74	76.26 ± 7.04∗∗	88.7 ± 6.72∗∗^##^	75.57 ± 12.07	64.12 ± 4.85^++^	78.97 ± 6.28^※※^
Vertical jump (cm)	52.16 ± 5.77	52.87 ± 5.84	50.1 ± 6.22	35.75 ± 6.72	36.21 ± 4.37	38.08 ± 4.89
Sit-and-reach (cm)	29.07 ± 5.25	24.26 ± 6.08∗∗	22.03 ± 7.24∗∗	27.18 ± 5.53	26.78 ± 5.7	25.61 ± 6.7
Pull-up (reps)	34.05 ± 4.49	37.62 ± 5.18∗∗	34.14 ± 7.78^#^	19.3 ± 10.98	21.76 ± 9.69	17.23 ± 12.54
3 km run (s)	701.19 ± 30.95	715.75 ± 44.96	706.6 ± 51.94	835 ± 44.56	827.59 ± 68.56	848 ± 57.12
Cycling peak power out (W)	1101.9 ± 174			639.6 ± 138.9		
Cycling mean power out (W)	641.7 ± 68.7			400.5 ± 54.7		

Note: In male group, ∗significant difference compared with the Laser sailors (*p* < 0.05), ∗∗significant difference compared with the Laser sailors (*p* < 0.01). ^#^significant difference compared with the male 470 helmsmen (p < 0.05), ^##^ significant difference compared with the male 470 helmsmen (*p* < 0.01).

In female group, ^+^significant difference compared with the Laser Radial sailors (*p* < 0.05), ^++^significant difference compared with the Laser Radial sailors (*p* < 0.01). ^※^significant difference compared with the female 470 helmsmen (*p* < 0.05), ^※※^ significant difference compared with the female 470 helmsmen (*p* < 0.01).

Table 3 shows the multiple linear regression analysis for Laser sailors using the forward stepwise method. This model had a linear relationship of 82.7% and a goodness of fit of R^2^ = 0.655. The equation for this model was as follows: performance = 90.963-1.33 × sailing experience-0.461 × bench press-0.018 × cycling peak power out. The regression effect of equation is significant by statistical test, the Student's T test check the prediction value of the performance obtained with this equation, the result showed that the variables sailing experience, bench press and cycling peak power out are the ones that will determine the performance with a positive relationship (*p* < 0.01).

**Table 3:** Coefficients of the multiple linear regression model using the forward stepwise method for Laser sailors.

Model		Non-Standardized Coefficient		Typified Coefficient	t	Sig.	Collinearity Statistics	
		B	Std. Error	Beta			Tolerance	VIF
3	Constant	90.963	13.314		6.832	0.000		
	Sailing experience	−1.330	0.241	−0.560	−5.522	0.000	0.905	1.105
	Bench press	−0.461	0.132	−0.354	−3.5	0.001	0.911	1.097
	Cycling peak power out	−0.018	0.006	−0.289	−2.983	0.005	0.990	1.010

Method used: forward stepwise. B = linear regression coefficient; Std. Error = estimated error; t = statistical significance; Beta = standardized partial regression coefficient; Sig = level of significance, VIF = variance inflation factor.

Table 4 shows the multiple linear regression analysis for Laser Radial sailors using the forward stepwise method. This model had a linear relationship of 89.6% and a goodness of fit of R^2^ = 0.767. The equation for this model was as follows: performance = 27.433-0.391 × sailing experience+0.351 × vertical jump-0.329 × pull-up-0.027 × cycling peak power out. The regression effect of equation is significant by statistical test, the Student's T test showed that the variables sailing experience, vertical jump, pull-up and cycling peak power out are the ones that will determine the performance (*p* < 0.01).

**Table 4:** Coefficients of the multiple linear regression model using the forward stepwise method for Laser Radial sailors.

Model		Non-Standardized Coefficient			Typified Coefficient	t	Sig.	Collinearity Statistics	
		B	Std. Error		Beta			Tolerance	VIF
4	Constant	27.433	4.413			6.216	0		
	Sailing experience	−0.391	0.185	−0.271		−2.12	0.045	0.549	1.821
	Vertical jump	0.351	0.153	0.297		2.297	0.031	0.535	1.869
	Pull-up	−0.329	0.092	−0.455		−3.591	0.002	0.557	1.795
	Cycling peak power out	−0.027	0.008	−0.467		−3.374	0.003	0.467	2.14

Method used: forward stepwise. B = linear regression coefficient; Std. Error = estimated error; t = statistical significance; Beta = standardized partial regression coefficient; Sig = level of significance, VIF = variance inflation factor.

## 4 Discussion

The aim of the current study was to investigate the differences in the physical fitness characteristics of Chinese single-handed dinghy and 470 sailors, predict the single-handed dinghy sailors’ physical factors on the performance by equation to help us formulate training program.

Sailing is a height-dependent and weight-dependent activity, height and weight directly affect the righting moment of hiking and performance.^11^^,^^19^ Specific body weight ranges allow for a better balance the friction between the boat and the water as well as the force of the wind on the sail.^20^ The influence of height is mainly reflected in that sailors will use themselves as the righting moment to counter the heeling moment generated by the wind during hiking, the upper body as the moment arm largely depends on the height.^19^^,^^21^ The results of this study showed that the 470 helmsmen are shorter, lighter and have a lower BMI, 470 crew are similar in height to the single-handed dinghy sailors, but lower in weight and BMI. The single-handed dinghy and 470 class boats weigh 59 kg and 120 kg respectively, that optimal requirements differ between different sailing classes and between the helmsmen and crew. The 470 class has a crew of two where the helmsmen hikes and the crew trapezes, the crew is usually taller and heavier than helmsmen, because they hang out the side of the boat on a trapeze and have a greater influence on the righting moment of the boat. Bojsen-Moller also found that hikers are heavier and have a higher BMI than crew. In the case of light wind, sailors pay more attention to sailing skills and route selection, and a lighter weight is beneficial to reduce the sailing resistance. However, the increase of the force acting on the sail will increase the heeling moment when the wind is strong, higher righting moment is needed correspondently.^22^ Under the condition of similar height, people with light weight have smaller righting moment, which increases the demands on physical fitness and makes them easier to fatigue. Therefore, it is common to have a sailor who presents a better performance in specific wind conditions, which is more adapted to their anthropometric characteristics.

Sailing regatta is a race around the mark in a certain sea area, each race goes through several rounds of upwind, downwind and crosswind legs, two third total racing time is spent on sailing upwind, which may vary with wind condition, sea state and race route setting.^20^^,^^23^ Upwind sailing is the stage with the highest physical demands on sailors, the side-deck hikers will be use hiking technique 94% of the time in winds more than 8 knots, and the trapeze sailors free pumping in winds above approximately 10 knots which clearly increases the physiological demands.^22^

**Fig. 1:**
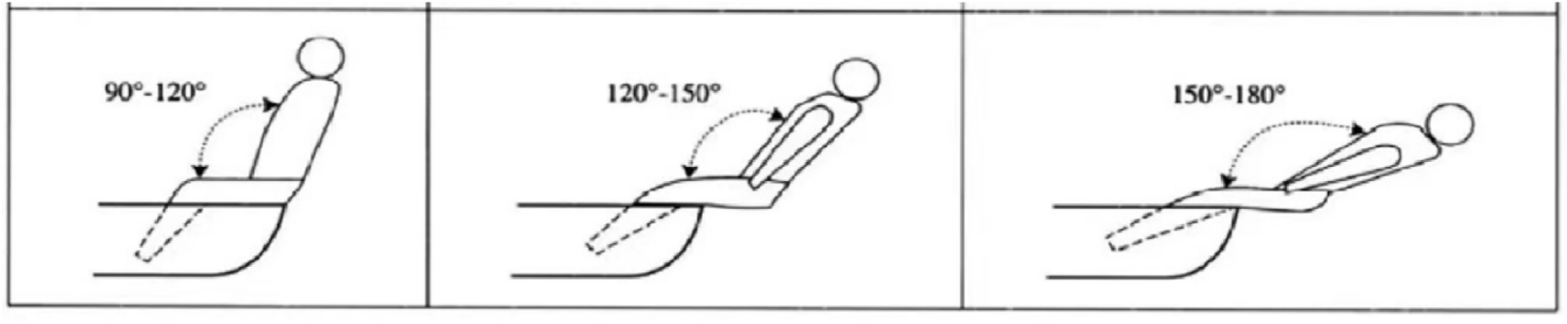
Classification of different hiking positions.^18^

Muscular strength is related to the force a muscle or muscle group can exert in one maximal effort, also called maximum strength.^16^ Greater muscular strength enhances the ability to perform general sport skills as well as decreases the risk of injury, but also improves an individual's performance.^24^ This study found that the strength of elite Laser sailors' lower body strength was higher than male 470 sailors, which was caused by the special body position and physical demands during hiking. Single-handed dinghy sailors hiking technique can be divided into three types according to different body angles (Fig. 1): (1) Siting hiking (90°–120° enclosed hip angle), (2) upright hiking (120°–150° enclosed hip angle) and (3) extended hiking (150°–180° enclosed hip angle).^18^ No matter what kind of hiking position produces great pressure on the lower body muscles, especially the quadriceps.^25^ When hiking, the quadriceps muscle produces the knee joint extension force with the aid of the hiking strap, the hip joint flexes and the body leans back. The larger the hip angle is, the higher the requirement for the strength of the lower body. Back squat is an exercise that is used to primarily strengthen the quadriceps and the gluteus, and the level of muscle activations is similar to those of hiking, good quadriceps muscle strength can optimize the maximum hiking performance, so back squat is the primarily essential in the training of Laser sailors.^26^ In the present study that 470 crew have better bench press and back squat strength than helmsmen. For one thing, 470 crew must rely more on muscles to provide support in trapeze sailing. For another, body pumping movements as the wind speed increased, trunk and knee extension also increased as a response to maintain the righting moment.^9^

The receptions of pull-up are commonly used to assess forearm and upper arm flexor strength endurance.^27^ When sailing upwind, the arms do constantly flexion and extension movement, adjust the sail and mainsheet in order to preserve boat balance. The sailing downwind requires less physical demand for sailors, but more attention should be paid to the arm and shoulder strength endurance. Only by increasing the strength and range of sheeting the sail can make the boat produce the maximum speed when the force on the sail is strong, and if the sail does not have sufficient force, it will lead the boat's speed to slow down. At this time, it is necessary to push the rudder and tighten the sail quickly, so the boat can accelerate by the waves. During the whole race, the sailors have been in the state of sheeting the sail, so the upper body strength endurance is one of the specific training contents of the sailors. In this study, the upper body strength endurance of elite sailors was better which may be the result of long-term specific adaptation on water and physical fitness training on land. The weight of 470 helmsmen was obviously lighter and had less resistance to overcome, so the repetitions of pull-up under self-weight conditions were more than the singe-handed dinghy sailors and 470 crew.

Flexibility is the ability to move a joint through its complete range of motion. The ability to fully use one's range of motion plays a role in day-to-day activities and athletic endeavors.^28^ Improving the range of motion of the joints allows for smoother movement and contributes to more effective exercise. The sit-and-reach is one of the most common tests of flexibility, which is used to evaluate the flexibility of the lower back and hips.^29^ Previous studies have shown that elite Laser sailors have good flexibility of lower body.^3^ In this study, the sit-and-reach ability of single-handed dinghy sailors was also better than 470 sailors. During single-handed dinghy sailing, the stability of the boat largely depends on the time of the sailor to perform the maneuvers, the hip flexion ability during hiking, the ability of hamstring stretching in tacking and gybing, in addition, Laser regatta has more intense racing conditions, good flexibility enables sailors to complete maneuvers more efficient and maintain a higher boat speed.^10^

The variables that were related to Laser performance were sailing experience, bench press strength, cycling peak power out. The equation obtained was 90.963-1.33 × sailing experience-0.461 × bench press strength-0.018 × cycling peak power out, which explained 65.5% of the performance of the Laser sailors. The variables that were related to Laser Radial performance were sailing experience, vertical jump, pull up, cycling peak power out. The equation obtained was 27.433-0.391 × sailing experience+0.351 × vertical jump-0.329 × pull-up-0.027 × cycling peak power out, which explained 76.7% of the performance of the Laser Radial sailors.

According to Polato^30^, the experience of the sailors is determinant for performance, and high performance sport can only be reached when its basis is developed since childhood. Therefore, in this study, the sailing experience and physical factors were combined to analyze sailor's performance, and the results showed that the sailing experience had a certain predictive effect. Experienced sailors, who can change the course quickly and modify the speed and direction of the vessel through the tacking and gybing, delay the onset of fatigue and have better control the amplitude of the body movements compared to inexperienced sailors.^31^^,^^32^ From the perceptual area, sailing requires a high level of visual stimulus perception, more experienced sailors have a better ability to perceive external stimuli and extract relevant information from the environment and make decisions to facilitate the continuous adaptation to environmental conditions, generating visual and motor behavior adaptive pattern,^33^^,^^34^ quick decision making with appropriate and continual control inputs stimuli effectively determine sailing performance.^35^

In sailing race, the utilization of the wind is foundation, the physical fitness is key. Not only environmental conditions are crucial to the success for the sailors, but physical fitness as a controllable aspect also plays an important role in performance. In this study, there were differences in the physical fitness factors that predicted the performance of single-handed dinghy sailors, including anaerobic capacity and upper body strength for Laser, and anaerobic capacity, lower body power and upper body strength endurance for Laser Radial. The difference in physical fitness factors has a certain relationship with the equipment of difference classes, Laser and Laser Radial's boat type and weight are same, but sail area and mast length are different, Laser sail area is 7.06 square meters and Laser Radial is 5.76 square meters. The boat with a larger sail area carries more force, which requires more upper body strength to control the rope, especially in strong wind conditions. Laser Radial sailors don't require high-level upper body strength, but they constantly adjust the rope to sheet the sail requires greater strength endurance. Some studies have also shown that single-handed sailors have better upper body strength endurance compared with non-sailors.^36^ In addition, there is a greater sex-difference in upper compared to lower body strength in adults, because male upper body muscles tend to have more androgen receptors compared to lower body muscles, which may result in greater muscle development and strength.^37^^,^^38^

Anaerobic capacity is an important parameter for athletic performance, not only for short high-intensity activities but also for breakaway efforts and end spurts during endurance events.^39^ Our study examines the aspect of lower-body power and anaerobic capacity using the 30-s all-out exhaustive cycle test. The peak power out recorded is the maximal power output achieved for 5-s of the test, and the average power out is recorded and averaged over the entire 30-s of the test.^40^ The ﬁnding of the present study was that peak power out is one of the factors affect the performance of single-handed dinghy sailors. It is different with previous studies that found aerobic demand is signiﬁcantly more important for Laser sailors and blood lactate concentrations never reach very high level even at the end of hard hiking.^35^^,^^41^^,^^42^

One important issue cannot be ignored is that the oxygen intake of the sailors during sailing is kept at a low level, aerobic capacity is only used moderately and should not be overemphasized in training.^43^^,^^44^ Anaerobic capacity plays an more and more important role in the strong wind, especially in the hiking of the full range is opened, the motion of lower body quasi-isometric contraction to produce certain blood restriction, quadriceps’ muscle oxygen saturation decreases obviously, so in the process of single-handed dinghy sailing requirements high-level anaerobic capacity.^44^^,^^45^ Vangelakoudi^4^ suggests that mean and maximal anaerobic power and ranking of the nationally ranked sailors have strong correlations. When sailors suddenly pull the sheet, to contract to a significantly higher number of muscle cells than in the conditions of daily life, will be use a certain amount of energy from their anaerobic capacity.^7^

Muscular power is the rate of performing work or the product of force and velocity, athletes with a stronger power might have greater potential in sports where these attributes contribute to competition success,^46^^,^^47^ Laser sailors need to apply large forces to the hiking strap in order to maintain boat trim in choppy and gusty conditions. Vertical jump is one possible alternative that would be to estimate lower body power,^13^ it requires a combination of strength, speed, and power of muscles involved in hip extension (gluteus maximus), knee extension (the quadriceps femoris). Robertson showed that the propulsive phase for vertical jump, the contributions of the hip, knee, and ankle muscles were 40.0%, 24.2%, and 35.8%, respectively.^48^ At this time, the rapid coordinated force generation of knee joint and hip joint is similar to explosive hiking movement during the start, confrontation, and tacking of the single-handed dinghy. Although Laser Radial sailors are lighter, strong power helps them execute a series of maneuvers to change course or increase the boat speed when the boat is unstable in strong wind conditions.

## 5 Limitation and suggestion of the study

Since the 470 is double-handed dinghy with two people operating, the factors that affect the performance are not only the complex environment factors, but also the coordination between the sailors and the application of techniques and tactics. Therefore, the relationship between physical fitness and performance of the crew and helmsmen cannot be discussed separately. Future research should find an appropriate method to analyze the physical fitness and performance of the double-handed dinghy.

## 6 Conclusion

The 470 helmsmen are shorter, lighter and had a lower BMI, 470 crew are similar in height to the single-handed dinghy sailors, but lower in weight and BMI. Laser sailors have better strength and flexibility than the ones of male 470. There is no significant difference in the physical fitness characteristics between the Laser Radial and female 470 crew, but they both had better upper and lower body strength than female 470 helmsmen. The regression equation is possible to explain 65.5% of the performance of the sailors in Laser = 90.963-1.33 × sailing experience-0.461 × bench press-0.018 × cycling peak power out; The regression equation is possible to explain 76.7% of the performance of the sailors in Laser Radial = 27.433-0.391 × sailing experience+0.351 × vertical jump-0.329 × pull-up-0.027 × cycling peak power out. Performance in laser and Laser Radial sailors will be determined by the technique and tactics (sailing experience) as well as physical fitness.

## Author statement

Dandan Pan: Conceptualization, Methodology, Software, Writing- Original draft preparation.

Bingshu Zhong.: Data curation, Writing – Review.

Wei Guo: Visualization, Investigation.

Yixiao Xu: Writing – Review, Supervision.

## Declaration of competing interest

We declare that we have no financial and personal relationships with other people or organizations that can inappropriately influence our work, there is no professional or other personal interest of any nature or kind in any product, service and/or company that could be construed as influencing the position presented in, or the review of，the manuscript entitled “Physical fitness characteristics and performance in single-handed dinghy and 470 classes sailors”.
